# A missense variant in *EXOSC8* causes exon skipping and expands the phenotypic spectrum of pontocerebellar hypoplasia type 1C

**DOI:** 10.1038/s10038-023-01207-4

**Published:** 2023-11-29

**Authors:** Maha S. Zaki, Sherif F. Abdel-Ghafar, Mohamed S. Abdel-Hamid

**Affiliations:** 1 Clinical Genetics Department, Human Genetics and Genome Research Institute, National Research Centre, Cairo, Egypt; 2 Medical Molecular Genetics Department, Human Genetics and Genome Research Institute, National Research Centre, Cairo, Egypt

**Keywords:** Neurological disorders, Neurodegeneration

## Abstract

Pontocerebellar hypoplasia (PCH) is a rare heterogeneous neurodegenerative disorder affecting the pons and cerebellum and is currently classified into 17 types (PCH1-PCH17). PCH1 is distinguishable from other types by the association of spinal motor neuron dysfunction. Based on the underlying genetic etiology, PCH1 is further classified into 6 different subtypes (PCH1 A-F). Of them, PCH type 1C is caused by pathogenic variants in *EXOSC8* gene and so far, only four families have been described in the literature. In this study, we report a new patient with PCH1 who proved by whole-exome sequencing to harbor a novel homozygous missense variant in the splice region of *EXOSC8* gene (c.238 G > A; p.Val80Ile). Studying mRNA of the patient confirmed that this variant results in skipping of exon 5 of the gene and early protein truncation. Our patient presented with the main clinical findings of PCH type 1C including psychomotor retardation, spasticity, spinal muscle atrophy, and respiratory problems. However, unlike most of the reported cases, he did not develop hearing or visual impairment and displayed a longer survival. In addition, our patient had dysmorphic facies, nystagmus, congenital esotropia and contractures which were infrequently described in patients with *EXOSC8*. Diaphragmatic hernia, dilated lateral ventricles, hypoplastic temporal lobes, and thinning of the brain stem were additional new findings noted in our patient. This study presents the fifth family with this extremely rare type of PCH and expands the associated clinical and brain imaging findings.

## Introduction

The RNA exosome is an evolutionarily conserved, multi-subunits ribonuclease complex that is critical for processing and/or degrading a variety of RNA molecules [1]. Eukaryotic RNA exosome consists of a two layered ring like structure made from nine conserved core subunits, through which RNAs could pass. The upper ring (also known as cap of the exosome) is composed of EXOSC1-3 while the lower ring (the barrel of the exosome) is made by EXOSC4-9. The human exosome had two additional catalytic subunits “EXOSC10 and DIS3” [2, 3]. The RNA exosome functions in both the nucleus and the cytoplasm [4, 5]. Inside the nucleus, the exosome processes and degrades multiple RNA precursors such as un-spliced pre-messenger RNAs and cryptic transcripts while in the cytoplasm, it targets RNAs with AU-rich elements (AREs) or RNAs that escaped nucleus degradation [3].

Recently, pathogenic variants in six exosome subunits genes have been associated with different tissue specific disorders [3]. Biallelic variants in *EXOSC3, EXOSC8, EXOSC9,* and *EXOSC1* have been reported in different subtypes of pontocerebellar hypoplasia type 1 (PCH1), which is usually associated with spinal motor neurons dysfunction [6–9]. On the other hand, *EXOSC2* variants cause a recessive neurological disease with short stature, hearing loss, retinitis pigmentosa, and distinctive facies (MIM #617763) [10, 11]. Furthermore, dominant variants in *DIS3* have been linked to multiple myeloma [12].

To date, only four families with PCH type 1C have been described in the literature [7, 13]. Herein, we describe an additional family with PCH type 1C harboring a novel missense variant in *EXOSC8* gene. In addition, we present a review of all reported cases to refine the clinical and brain imaging findings of the disorder.

## Patients and methods

### Patient

The Neurogenetics/Neuropediatrics Clinic at the National Research Centre (NRC), Cairo received a referral for diagnosis and counseling of a 3 years old boy from Algeria. Thorough medical history, pedigree construction, and full general and neurological assessments were conducted. Investigations including karyotyping, metabolic work up, ophthalmological evaluation, neurophysiological studies (EEG, electromyography and nerve conduction velocity, visual evoked potential, electroretinogram, auditory brain stem evoked potential), and neuroimaging were performed.

The Medical Research Ethics of the NRC in accordance with “World Medical Association Declaration of Helsinki” in 1995 (as revised in Seoul 2008) approved the research study (Approval: 20105) and written informed consent was obtained from the parents.

### Molecular analysis

#### Whole exome sequencing (WES) and bioinformatic analysis

Genomic DNA from the patient and his parents was extracted from peripheral blood samples using Qiagen Blood DNA Kit (Qiagen, Hilden, Germany) and quantified by a Nanodrop 2000 system (Thermal Fisher Scientific, Inc., Waltham, Massachusetts, USA). WES was performed using SureSelect Human All Exome 50 Mb Kit (Agilent, Santa Clara, CA, USA) and analyzed on Illumina NovaSeq 6000 (Illumina, San Diego, CA, USA). The obtained sequences were aligned to UCSC human genome GRCh37/hg19 and variants were verified through the GATK pipeline. Identified variants were checked against public genetic databases like Genome Aggregation Database (gnomAD, https://gnomad.broadinstitute.org/), 1000 Genomes (www.1000genomes.org), and dbSNP (http://www.ncbi.nlm.nih.gov/SNP/). Pathogenicity of detected variants were predicted using various bioinformatics tools as SIFT (https://provean.jcvi.org/protein), PolyPhen-2 (https://genetics.bwh.harvard.edu/pph2/) and MutationTaster (https://www.mutationtaster.org/). Analysis of the raw data, variant annotation and prioritization were performed using the BaseSpace Variant Interpreter Server. Variants were prioritized based on the mode of inheritance, relation the patient’s phenotype, minor allele frequency and in silico predicted pathogenicity.

#### Segregation analysis

Segregation analysis of the identified variant in the parents and the healthy sib was conducted by PCR amplification of exon 5 of the *EXOSC8* using specific primer designed by Primer3 software followed by purification with Exo-SAP PCR Clean-up kit (Fermentas, Germany) and sequencing using the BigDye Terminator v3.1 Cycle Sequencing Kit (Applied Biosystems, Foster City, CA, USA) on the ABI Prism 3500 Genetic Analyzer (Applied Biosystems) according to manufacturer’s instructions.

#### Functional study of the c.238 G > A (p.Val80Ile) variant

To study the effect of the missense variant c.238 G > A (p.Val80Ile) on splicing, total RNA was isolated from patient’s cultured primary fibroblasts using QIAamp RNA kit (Qiagen, Hilden, Germany). Five µg of total RNA were reverse transcribed into cDNA using COSMO cDNA Synthesis Kit (Willowfort, Birmingham, United Kingdom). The synthesized cDNA was then used as a template for partial amplification of the *EXOSC8* gene (from exons 3 to 7) using one pair of primers: 5′-CCACAACTGTCAACATCGGT-3′ and 5′-CAGACAAGCTTTCCTGGAGA-3′ under the following conditions: 96 °C for 2 min, a total of 35 cycles of 94 °C for 30 s, annealing at 62.5 °C for 30 s, 72 °C for 30 s, and a final extension of 72 °C for 5 min. PCR products were separated by 1% agarose gel electrophoresis and then purified and sequenced as described above.

## Results

### Clinical evaluation

**Fig. 1:**
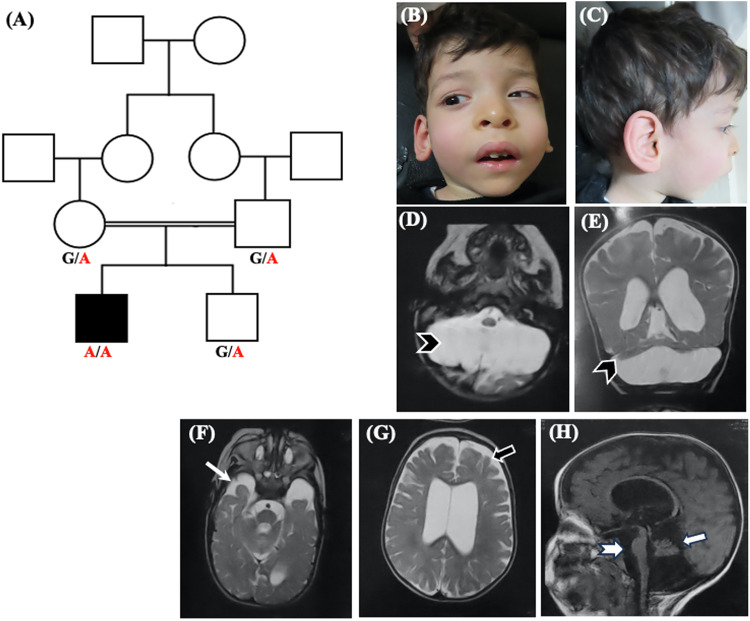
**A** Pedigree of the studied family. **B**, **C** Face and profile of our patient showing dolichocephaly, long face, sparse arched eye brows, hypertelorism, broad nasal root, downslanting palpebral fissures, esotropia, hypoplastic alae nasii, smooth long philtrum, prominent upper lip, receded mandible, pointed chin and low-set large ears. **D**–**H** Brain MRI, Axial T2W (**D**, **F**, **G**), Coronal T2W (**E**), and Sagittal T1W (**H**) showing severe hypoplasia of cerebellum (**D**, **E**; arrow head), hypoplastic temporal lobe (**F**; long arrow), mild cortical atrophy (**G**; short black arrow), dilated asymmetric lateral ventricles (**G**), hypoplastic vermis (**H**; white short arrow), mild brain stem hypoplasia (**H**, notched arrow) and thin corpus callosum (**H**)

Our patient is a 3 years and 4 months old boy born to a healthy first cousin couples from Algeria (Fig. 1A). He is the first child in the family and had a healthy younger brother. No similar family history was recorded. The pregnancy history was uneventful and delivery was by Cesarean section at the 39th week of gestation. Lethargy and abnormal breathing were noted few minutes after birth and he was admitted to NICU directly for 2 weeks. Anthropometric measurements at birth were verified as weight 3 kg (−1SD), height 49 cm (−0.5 SD), and head circumference 34.3 cm (−0.9 SD). During infancy and early childhood, recurrent vomiting and dyspnea were prominent manifestations owed to the presence of diaphragmatic hernia that was operated at the age of 16 months. Our proband did not achieve any developmental milestones except for fairly recognizing his parents and vocalized. He could not use the hands or respond to his name. Epilepsy was not noted by parents. On examination, the patient had quadriparesis and failed to support the head. His weight was 9 kg (−3.75 SD), height 87 cm (−2SD) and head circumference 48.7 cm (−0.9 SD). Craniofacial features showed dolichocephaly, long face, sparse arched eyebrows, hypertelorism, broad nasal root, downslanting palpebral fissures, esotropia, hypoplastic alae nasii, smooth long philtrum, prominent upper lip, receded mandible, pointed chin, microstomia, low-set large protruded ears and asymmetric larger right side (Fig. 1B). General examination was normal except for arthrogryposis, clenched hands and long toes.

Neurological evaluation showed severe axial hypotonia, limbs hypertonia and brisk reflexes, and positive Babinski sign. Investigations revealed normal karyotyping, metabolic screening, acylcarnitine profile, organic acids in urine, echocardiography, abdominal sonar, auditory brain stem evoked potential, electroencephalogram and fundus examination. EMG and NCV identified anterior horn cells dysfunction. Brain MRI revealed mild cortical atrophic changes, asymmetric dilatation of lateral ventricles more in the left side, thin corpus callosum, hypoplastic temporal lobes, severe hypoplasia of cerebellum (more involvement of the hemispheres) and mild brain stem hypoplasia (Fig. 1D–H).

### Molecular findings

**Fig. 2:**
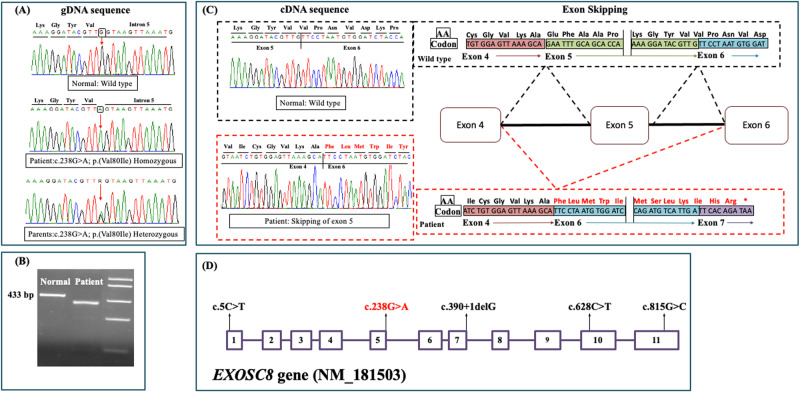
**A** Portions of the gDNA sequencing electropherograms showing the *EXOSC8* variant identified in our patient. The arrow indicates the site of variant. **B** A 1% agarose gel showing partial amplification of the cDNA of the *EXOSC8* (from exons 3 to 7) in our patient and a normal control subject. **C** Part of the sequencing electropherograms of the cDNA fragment showing exon 5 skipping. **D** Schematic diagram of the *EXOSC8* gene showing all reported variants and their location

WES analysis identified a new homozygous missense variant (c.238 G > A, p.Val80Ile) in the *EXOSC8* (NM_181503.2), which is associated with PCH type 1C as the likely cause of the patient’s phenotype. The identified variant affects a highly conserved amino acid residue and is located in the last nucleotide of exon 5 of the gene. Segregation analysis confirmed the presence of the variant in the homozygous state in the patient while both parents and the healthy sibling were found to be heterozygous (Fig. 2A). The c.238 G > A variant is not found in public genetic databases or our in-house database of over 1500 exomes and is predicted to be disease-causing by different bioinformatic tools. In addition, Alamut, SpliceAi, dbscSNV and MaxEntScan software predicted that this variant might influence splicing.

### Effect of EXOSC8 variant on patient’s RNA

As the identified missense variant is located in the splice region, we tested its effect on splicing. We partially amplified the *EXOSC8* gene in cDNA of the patient and a normal control individual. Agarose gel electrophoresis showed that the patient had a shorter band in comparison to the normal control (Fig. 2B) and then sequence analysis confirmed that this variant induced exon 5 skipping and ultimately production of an early stop codon at exon 7 (p.Val80Phefs*39) (Fig. 2C).

## Discussion

PCH represents a group of clinically and genetically heterogeneous neurodegenerative disorders with prenatal onset [14]. PCH1 is characterized by cerebellar hypoplasia along with degeneration of the bulbar and spinal motor neurons, which is identical to spinal muscular atrophy (SMA). Initial reports of PCH1 described it as a fatal disorder in neonates, with symptoms such as polyhydramnios, congenital contractures, respiratory failure, and severe muscle weakness [15, 16]. However, subsequent studies illustrated that the ventral pons could be spared, and patients might survive till puberty, with broadening of the clinical and neuroradiological spectrum of PCH1 [17, 18].

At present, PCH1 is further classified into six subtypes (PCH1A-F) based on the underlying causative genes. Of them, four are associated with variants in exosome genes: PCH1B is caused by *EXOSC3* [6], PCH1C by *EXOSC8* [7], PCH1D by *EXOSC9* [8], and PCH1F by *EXOSC1* [9]. On the other hand, PCH1A and PCH1E are caused by variants in *VRK1* and *SLC25A46* genes, respectively [19, 20].

PCH type 1C is an ultra-rare subtype of PCH, with only four reported families [7, 13]. The initial report by *Boczonadi and co-authors* [7] described three unrelated consanguineous families, two of them were of Hungarian Romanian origin who shared the same missense variant (c.815 G > C, p.Ser272Thr). The third family was of Palestinian descent and had a different missense variant (c.5 C > T, p.Ala2Val). Subsequently, in 2021 *Rodríguez-García and coauthors* [13] reported a Spanish family with three heterozygous *EXOSC8* variants including the same missense variant found in the Hungarian Romanian families (Table 1).

**Table 1:** Clinical, neurological and brain imaging findings of patients with pathogenic *EXOSC8* variants

	Bocozonadi et al. (2014)											Rodríguez-García et al.. (2021)	This study
	Family 1 (18 patients)							Family 2		Family 3		Family 4	Family 5
	Patient 1	Patient 2	Patient 3	Patient 4	Patient 5	Patient 6	Patient 7	Patient 1	Patient 2	Patient 1	Patient 2		
**Sex**	M	F	M	M	F	M	M	F	M	M	M	M	M
Consanguinity	+							-		+		-	+
Onset/death	2 m/ 11 m	2 m/ 9 m	4 m/ 13 m	1 m/ 14 m	1.5 m/ 18 m	12d/ 8 m	2 m/ 19 m	1.5 m/ 13 m	2 m/ alive 9 m	6 m/ 28 m	4 m/ alive 5 y	5 m/alive 16 y	Since early life/ alive 3 y
Facial dysmorphism	+	-	+	+	-	-	-	-	-	-	-	-	+
Ophthalmological abnormalities	Vision loss	Vision loss	Vision loss	Vision loss	Vision loss	Vision loss	Vision loss	Vision loss	Vision loss	-	-	Congenital esotropia, Nystagmus (5 m) Ophthalmoparesis, Optic disk drusen.	Congenital esotropia, nystagmus
Hearing loss	+	+	+	+	+	+	+	+	+	-	-	-	-
Motor dysfunction	+	+	+	+	+	+	+	+	+	+	+	+	+
Respiratory problems	+	+	+	+	+	+	+	+	+	+	+	+	Recurrent chest infection, after birth abnormal breathing. tachypnea
Neurological signs													
Muscle weakness	+	+	+	+	+	-	-	+	+	+	+	+	+
Spasticity	Spastic tetraparesis	Spastic tetraparesis	+	Spastic tetraparesis	Spastic tetraparesis	Spastic tetraparesis	Spastic tetraparesis	Spastic tetraparesis	Spastic tetraparesis	-	-	Lower limbs spasticity	Spastic tetraparesis
Hypotonia	-	-	+	+	+	-	-	-	-	-	-	-	Severe axial hypotonia and limbs hypertonia
Contractions	-	-	-	+	+	-	-	-	-	+	-	-	+
MRI findings	N/A	2.5 m	5 m	11 m	2 m	N/A	6 m	N/A	5 m			2 y	2 y
Cortical atrophy		+	-	+	-		+		-	-	-	-	+
Cerebellar atrophy		-	-	+	-		-		-	+	+	+	+
Vermis hypoplasia		+	+	-	-		-		-	+	+	+	+
Thin corpus callosum		+	-	+	+		-		+	-	-	-	-
Others		-	-	-	Immature myelination		-		Immature myelination	Mega cisterna magna	Mega cisterna magna	-	Dilated third ventricles, mild thinning of brain stem, hypoplastic temporal lobes
Other abnormalities	Inguinal hernia Scoliosis	Tremor	Inguinal hernia	Brachycephaly, Tremor	Tremor	Tremor	TremorFeeding difficulties	Inguinal herniaFeeding difficulties	Feeding difficulties	Feeding difficulties	Feeding difficulties	Dysmetria Dysdiadochokinesia	Diaphragmatic hernia operated at 14 months, long toes.
Ethnicity	Hungarian Romanian							Hungarian Romanian		Palestinian		Spanish	Algerian
Variant	c.815 G > C							c.815 G > C		c.5 C > T		c.390+1delG/c.618 C > T/c.815 G > C.	c.238 G > A
Effect on protein	p.Ser272Thr							p.Ser272Thr		p.Ala2Val		p.Ser116Lysfs*27/p.Pro210Ser/ p.Ser272Thr.	p.Val80Ile

Family 1 of *Boczonadi* et al. [7] comprised 7 patients, who all died with respiratory failure before reaching their second year. Similarly, one of the two sibs of Family 2, with the same variant, died at the age of 13 months while the other sib was alive at the time of publishing their study (9 months). The two sibs of Family 3 harboring the c.5 C > T (p.Ala2Val) variant showed a longer survival as one died at the age of 28 months while the other was alive (5 years). This led *Boczonadi and co-authors* [7] to consider PCH type 1C as a lethal type of PCH. Diversely, *Rodríguez-García* et al. [13] reported a 16-year-old boy with compound heterozygous variants in *EXOSC8* and a slowly progressive milder phenotype. Our patient was 3 years and 4 months old at his last examination that pointed to better longevity in comparison to most of the reported cases.

Almost all described patients with *EXOSC8* variants including ours (*n* = 13) exhibited psychomotor retardation, spasticity, SMA, and respiratory problems. The respiratory issues are likely caused by deficiencies in the respiratory chain complexes resulting in symptoms such as recurrent chest infections, tachypnea, abnormal breathing patterns which could eventually lead to respiratory failure [7, 13]. Spasticity was noted in 11/13 patients and was affecting both upper and lower limbs except for one patient who had lower limbs spasticity [7]. Impairment of hearing and vision were detected in all patients with the p.Ser272Thr variant described by *Boczonadi* et al. [7]. In contrast, Family 3 of *Boczonadi* et al. [7], the single patient described by *Rodríguez-García* et al. [13], and our patient showed normal hearing and did not develop visual loss. Interestingly, eye examination of our patient showed nystagmus and congenital esotropia which were also present in the single patient described by *Rodríguez-García and co-authors* [13].

Facial dysmorphism does not appear to be a unique feature for patients with *EXOSC8* as only three patients (3/12) had specific facial features without clear description of such features [7]. Our patient had characteristic facies including dolichocephaly, long face, hypertelorism, receded mandible, pointed chin, microstomia and low-set large protruded ears. Thoroughly investigation of the exome data of our patient didn’t show any related variants to the abnormal facial features. Therefore, we postulate that dysmorphic facies might be part of the phenotypic spectrum of *EXOSC8*-related PCH1.

Inguinal hernia was observed in three patients with PCH1C [7]. Interestingly, our patient exhibited a diaphragmatic hernia. Although there is no clear association yet between *EXOSC8* genes and the development of hernias, it could be attributed to the weak abdominal wall due to the severe axial hypotonia. Other rare findings with *EXOSC8* variants are feeding difficulties (5 patients), tremors (4 patients), contractures (4 patients), dysmetria (1 patient), dysdiadochokinesia (1 patient), brachycephaly (1 patient), and scoliosis (1 patient). The variability in symptoms observed among the reported cases strongly suggests that EXOSC8 is likely to affect multiple systems in the body, and additional research is needed to fully understand the underlying mechanisms and delineate the associated clinical manifestations.

The reported *EXOSC8* patients displayed variabilities in their brain imaging such as cerebellar hypoplasia, pontine hypoplasia, cerebral atrophy and thinning of the corpus callosum [7, 13]. Our patient had severe cerebellar hypoplasia (mainly hemispheres), mild cortical atrophic changes and thin corpus callosum. In addition, he had dilated ventricle, hypoplastic temporal lobes, and thinning of the brain stem which were not reported before extending the neuro-radiological spectrum of the disorder. Of note, the hemispheric cerebellar involvement in our patient was much more pronounced in comparison to the published scans.

In this study, we identified a new missense *EXOSC8* variant (c.238 G > A, p.Val80Ile) which was confirmed by studying mRNA of the patient to result in skipping of exon 5 and production of an early stop codon (p.Val80Phefs*39). Our new variant raises the total number of reported *EXOSC8* variants to five including four missense and one splice site variant (c.390 + 1delG) (Fig. 2D). Interestingly, this splice variant caused exon 7 skipping and also resulted in early protein truncation (p.Ser116Lysfs*27).

In conclusion, we described a new family with PCH type 1C and identified a novel *EXOSC8* missense variant that resulted in exon skipping. Although majority of the clinical findings of our patient were similar to the previously reported patients, new and unusual findings such as dysmorphic facies, nystagmus, congenital esotropia, contractures and diaphragmatic hernia were observed. In addition, brain MRI showed dilated ventricles, hypoplastic temporal lobes, and thinning of the brain stem, which were not detected before in patients with *EXOSC8* variants. Therefore, we believe that our study refines and expands the phenotypic and mutational spectrum of PCH type 1C.

## Data Availability

The data supporting the findings of this study are available with the corresponding author upon request.
